# A novel training device for tip control in colonoscopy: preliminary validation and efficacy as a training tool

**DOI:** 10.1007/s00464-017-5617-7

**Published:** 2017-06-07

**Authors:** Stephan Riek, Andrew Hill, Annaliese M. Plooy, Mark S. Horswill, Alanna St. G. Cresp, Welber Marinovic, Melany J. Christofidis, Robin Burgess-Limerick, Guy M. Wallis, Marcus O. Watson, David G. Hewett

**Affiliations:** 1 School of Human Movement Studies, The University of Queensland, Brisbane, Australia; 2 Clinical Skills Development Service, Metro North Hospital and Health Service, Brisbane, Australia; 3 School of Psychology, The University of Queensland, Brisbane, Australia; 4 School of Medicine, The University of Queensland, Brisbane, Australia

**Keywords:** Colonoscopy, Training, Skill assessment, Tip control, Motor skill

## Abstract

**Background:**

Effective control of the colonoscope tip is one of the most fundamental components of colonoscopy skill. Mastering fine tip control can be problematic for novice trainees, yet no validated training regimes exist for developing this specific skill component in isolation. We aimed to conduct a preliminary validation of a novel training device for colonoscopic tip control, and to assess its efficacy as a training tool.

**Methods:**

In study 1 (validation), 13 experienced colonoscopists and 16 novices used a colonoscope to accurately track 28 targets on each of four concave “training surfaces” as quickly as possible, and we compared their performance. In study 2 (pre–post-training study), another 16 novices were tested before and after a six-session training program. In both studies, the main outcome measurements were *completion time* (measured automatically by the device) and *variability of individual performance* (the *SD* of each individual’s completion times across trials).

**Results:**

Compared with novices, experienced colonoscopists were faster (*P* < 0.0001) and their performances less variable (*P* < 0.0001). With training, novices became faster (*P* < 0.0001) and more consistent (*P* = 0.003), and these improvements also generalized to novel training surfaces (*P*’s < 0.01). After training, the novices’ tip control performance was indistinguishable from that of the experienced colonoscopists (*P’s* > 0.05). The composite measures of *completion time* used in both studies all had acceptable to excellent internal consistency reliability (α’s ranged from 0.72 to 0.93).

**Conclusions:**

We found that performance measures derived from using the device to assess skill can discriminate between experienced colonoscopists and novices in terms of their ability to control and guide the colonoscope tip precisely, providing preliminary evidence to support the construct validity of the metrics. The device is also an effective training tool for this fundamental component of colonoscopy skill.

Colonoscopy is a complex cognitive-perceptual-motor task that is challenging to learn [1–3], and it is unclear what volume of procedures a trainee must perform to attain competence [4–6]. As with many complex clinical skills [7], colonoscopy skill can be decomposed into its elemental components [3, 7, 8]. Hence, one approach to developing early competence is the use of part-task trainers (i.e., devices that simulate a subset of the skill components in isolation [9]) to facilitate efficient motor skill learning by reducing the initial difficulty of the task for trainees [8]. Such devices allow beginners to engage in extensive practice of specific fundamental skills, with the aim of achieving automaticity before complexities are introduced that might otherwise cause cognitive overload and slow learning [7]. Part-task simulation training may be a beneficial learning strategy, both in terms of reducing the time it takes for trainees to achieve competence in the full task, and in lowering the inherent risks to patients associated with training via the traditional Halstedian apprenticeship model [7–9].

One of the most fundamental components of colonoscopy skill is effective control of the colonoscope tip, yet mastering the fine tip control required for all but the most rudimentary maneuvers can be problematic for trainees [2]. Efficient insertion of the colonoscope to cecum, thorough inspection of the mucosa, and therapeutic procedures such as polypectomy, all rely on highly developed tip control ability. While the importance of tip control in colonoscopy is well recognized [2, 3], no validated training regimes exist that are specifically designed to accelerate the early development of this important skill in isolation, and it remains difficult to master. One inherent difficulty is that the task requires the trainee to learn a complex visuomotor mapping between movements of the colonoscope shaft and angulation controls, and observed motion on the monitor. In situations such as this, where the visuomotor environment is arranged so that the direct links for control of limb movement are disrupted, there will be a breakdown in perceptual-motor speed and efficiency [10]. With effective practice, however, the learner can adapt to novel visuomotor mappings and retain the learned mental representations [11]. Hence we developed a novel tip control training device designed to optimize acquisition of this fundamental colonoscopy skill. The purpose of the studies presented here was to conduct a preliminary validation of the device, and to assess its efficacy as a training tool.

## Materials and methods

We conducted two studies. The first was a preliminary validation study in which we sought to establish that metrics derived from using the tip control training device could discriminate between experienced colonoscopists and novices (i.e., two groups that we would expect to perform differently if the performance metrics do in fact measure colonoscopic tip control skill). This is a commonly used technique for generating preliminary evidence that metrics derived from using a particular simulation device to assess performance of a skill have construct validity (i.e., they measure what they purport to measure [12–15]). In the second study, another group of novices were subjected to a brief training program comprising six 45 min sessions, and their tip control performance was assessed before and after to evaluate the efficacy of the device as a training tool. The research was approved by the Human Research Ethics Committee of The University of Queensland.

### Participants

Using G*Power 3.1.2 [16], a power analysis was conducted to determine the minimum sample size required to compare two groups using non-parametric Mann–Whitney *U* tests. Based on an expected experienced–novice difference of at least one pooled standard deviation, G*Power indicated that a minimum total sample of 28 participants was required for 80% power, with alpha set at 0.05 (one-tailed). An additional power analysis indicated that, to evaluate within-subjects training effects using non-parametric Wilcoxon signed-rank tests, only 9 participants were required for an equivalent level of power.

A convenience sample of thirteen experienced colonoscopists (gastroenterologists, *n* = 10; general physicians, *n* = 2; nurse endoscopists, *n* = 1) and sixteen colonoscopy novices participated in the preliminary validation study (study 1), and an additional sixteen novices took part in the training study (study 2). On average, the colonoscopists had 13.54 years of experience in endoscopic practice (range, 4–25; *SD* = 7.42), including 7173 colonoscopies (range, 1000–30,000; *SD* = 7543). They were all right handed except for one, who was ambidextrous. The novices were medical students in either their first or second year of study at The University of Queensland, who were all right handed and had no prior experience with colonoscopy. All participants were recruited and tested between September and October 2010, and gave informed consent.

### Tip control training device and associated general procedures

The prototype tip control training device evaluated in this paper was conceived and designed by one of the authors (AMP), with additional design contributions from several others (AH, MSH, WM, DGH, GMW, MOW, and SR). Engineering assistance was provided by staff from the EPSA Physics Mechanical Workshop at the University of Queensland, who also fabricated the plastic custom components. Each of these was made of precision-milled or precision-lathed polyvinyl chloride. Two authors (WM & AMP) developed custom pattern recognition software for use in conjunction with the physical device, using LabVIEW 7.1 with the Vision Development Module add-on (National Instruments, Austin, TX).

**Fig. 1:**
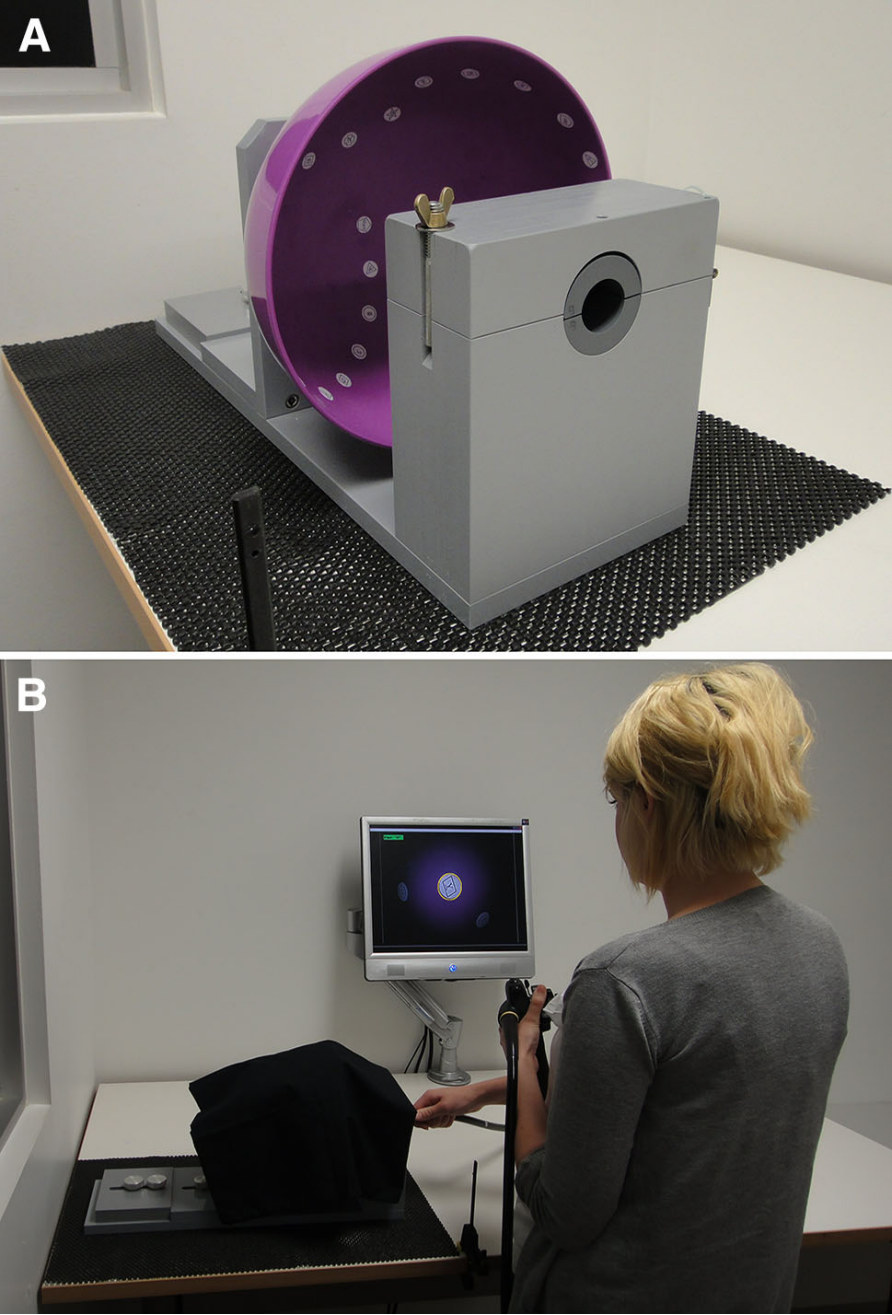
The tip control training device: in close up, showing the base, a concave training surface, and the colonoscope clamp (**A**); and in use with a standard colonoscope and custom software (**B**)

The tip control training device (Fig. 1A) has three main physical components: (1) a series of interchangeable hemispherical concave *training surfaces* (diameter = 220 mm); (2) a base to which one of the training surfaces can be securely attached; and (3) a clamp, built into the base, designed to hold a standard colonoscope with its tip at a fixed distance from the training surface (approximately 30 mm) without impeding tip flexion or torque steering. The clamp, which fixes the colonoscope in place at the 20 cm depth marker, prevents the shaft from being moved forwards or backwards but allows the colonoscope to rotate along its long axis.

In both of our studies, the device was presented atop a height-adjustable desk that was always set to 50% of the participant’s standing height (i.e., in proportional terms, the mean level chosen by experienced colonoscopists in a prior study [13]). Interposed between the device and the desk was a thin sheet of non-slip rubber with very low force-absorptive qualities, which prevented the device from moving across the desktop when force was applied to the colonoscope (Fig. 1A).

Seven different training surfaces were used in the studies reported here. Six of the surfaces were labeled A through F and the seventh, a practice surface, was labeled P. Each training surface contained a sequence of 28 circular “targets” (diameter = 10 mm), spaced approximately 30 mm apart (center-to-center), in a unique arrangement that formed a sequential trail (see Fig. 1A for an example). Within each trail, the targets were labeled “Start,” “A” to “Z,” and “End.” Each label also incorporated a unique geometric figure to assist with computer-based pattern recognition. Note that the targets were not intended to represent pathology. Rather, their purpose (achieved in conjunction with the pattern recognition software) was to standardize the precise positioning of the colonoscope tip required of each participant. A researcher was responsible for changing the training surface when required by the study procedures, and for covering the apparatus with a black cloth to occlude the colonoscope tip and ensure that the participant could not see the training surface directly (Fig. 1B).

The tip control training device was used in conjunction with an Olympus endoscopy system (Exera II CLV-180 light source, CV-180 video processor, and CF-H180DL colonoscope; Olympus Medical Systems Corp., Tokyo, Japan). Video from the processor was relayed to a laptop computer and sampled using the custom pattern recognition software, which supplied the endoscopic images to the participant’s monitor (Samsung LA22A450). This software also superimposed a yellow circle outline in the center of the screen to demarcate a “target zone” that had approximately the same on-screen diameter as the targets themselves (Fig. 1B).

In each trial of each study, the participant’s task was to manipulate the colonoscope using a combination of tip flexion via the angulation controls and torque steering, to bring each of the 28 targets into the target zone in sequential order, as quickly as possible. Each time a target entered the zone, the pattern recognition software identified it. Once the correct target was centered in the zone for 25 ms, the software changed the yellow target circle to red and generated a tone. This signaled to the participant that they should move the tip immediately to the next target (or that the trial was complete, in the case of the “End” target). The software also measured and recorded how long it took the participant to acquire the targets.

### Preliminary validation study procedure (study 1)

Each participant took part in a single data collection session, and was tested individually. All testing was conducted in a research laboratory in which the equipment was arranged to simulate an endoscopy procedure room. Prior to using the tip control device for the first time, novices watched a video that provided basic background information about colonoscopy and the colonoscope, including instructions on the use of the control dials. A second video, which explained and demonstrated “torque steering,” was followed by a practical demonstration of this technique by the researcher supervising data collection.

Before testing, participants in both groups received instructions explaining the novel tip control device and the associated general procedures, as described above. To promote torque steering, they were asked to keep their right hand on the colonoscope at around the 40 cm depth marker throughout the task, and to manipulate the control wheels with their left hand only.

Participants were allowed one practice trial using training surface P, before being tested once each on surfaces A, B, C, and E. These were presented in four different orders (ACBE, AEBC, BCAE, or BEAC) to which participants were randomly assigned using a list (created prior to data collection) where each option occurred four times in a random sequence generated in Microsoft Excel (Microsoft Corporation, Redmond WA). A copy of the same list was used for each group, and each participant was assigned the next available order in the sequence.

### Training study procedure (study 2)

A pre/post-test design was employed to quantify performance improvements following a course of tip control training sessions using the device. Each participant attended the research laboratory individually for sessions on 8 separate days, 2 per week, over the course of 4 consecutive weeks. Session 1 contained the pre-test. The following six sessions (sessions 2–7) comprised the training program. Finally, session 8 contained the post-test.

#### Pre-test (session 1)

Prior to the pre-test itself, participants viewed the same background information videos and received the same torque steering demonstration, task instructions, and practice trial as in the preliminary validation study. Participants were then tested twice on either training surface A (*n* = 8) or B (*n* = 8), according to random assignment.

#### Training (sessions 2 to 7)

In each of the six training sessions, participants completed the same set of exercises. Specifically, they practiced four different surfaces twice each in the following order: CCDDEEFF. Each training session lasted approximately 45 min.

#### Post-test (session 8)

In the post-test session, participants were tested on two different training surfaces, twice each. To assess training effects, participants were re-tested on the training surface that they had completed at pre-test (A or B). They were also tested on the alternative training surface (B or A), which they had not previously encountered, as a transfer test to assess whether their tip control skill would generalize to novel trails.

As in study 1, each participant was randomly assigned to one of four different orders using a random sequence generated in Microsoft Excel (Microsoft Corporation, Redmond WA) prior to data collection. Equal numbers of participants were assigned to complete the pre- and post-test trials in each specific order (Pre: AA Post: AABB, Pre: AA Post: BBAA, Pre: BB Post: AABB, Pre: BB Post: BBAA). This ensured that the design was counterbalanced such that: (a) half of the participants completed the post-test before the transfer test (and vice versa); and (b) each training surface served as the post-test (or transfer test) for an equal number of participants.

### Data scoring

#### Completion time

In both studies, the primary measure of performance was the time taken to acquire all targets along the trail. For each participant in the preliminary validation study, *completion time* was averaged across the four substantive trials. For the training study, separate average *completion times* were calculated for each novice’s performance on the pre-, post-, and transfer tests involving training surfaces A and B. Using a subset of the data from the validation study, we also calculated each experienced colonoscopist’s average *completion time* on these two surfaces, to compare against the trainees’ post-training performance.

#### Variability of individual performance

In both studies, we also assessed the extent to which individual participants performed the task consistently across multiple trials. For each of the *completion time* averages described above, we generated a corresponding variability score for each participant by calculating the standard deviation of their completion time scores across the relevant trials.

### Statistical analyses

All statistical analyses were conducted using IBM SPSS Statistics 19 (SPSS Inc., Chicago, IL), and alpha was set at 0.05. In both studies, we used Cronbach’s coefficient α to assess the internal consistency of the *completion time* measures (each of which was a composite formed by averaging over multiple trials, as described above). Cronbach’s α estimates scale reliability from the intercorrelations between responses to component items [17, 18]. Respectively, values equal to or exceeding 0.7, 0.8, and 0.9 may be regarded as indicating acceptable, very good, and excellent internal consistency [18, 19]. All of the subsequent analyses described below were conducted separately for each outcome measure: *completion time* and *variability of individual performance*.

For the preliminary validation study (study 1), the performance of experienced colonoscopists and novices was compared using Mann–Whitney *U* tests. For the training study (study 2), Wilcoxon signed-rank tests were used to compare trainees’ performance between: (a) the pre-test and the post-test (i.e., the same training surface pre- and post-training); (b) the pre-test and the transfer test (i.e., different training surfaces pre- and post-training); and (c) the post-test and the transfer test (i.e., different training surfaces post-training). Further Mann–Whitney *U* tests were used to compare the performance of the trainees at post-test with that of the experienced colonoscopists from the validation study.

For each significant difference between groups or tests, we calculated *r* as the measure of effect size [20]. Values of 0.30 and 0.50 can be regarded as indicating medium and large effects, respectively [21, 22].

## Results

### Preliminary validation study (study 1)

**Fig. 2:**
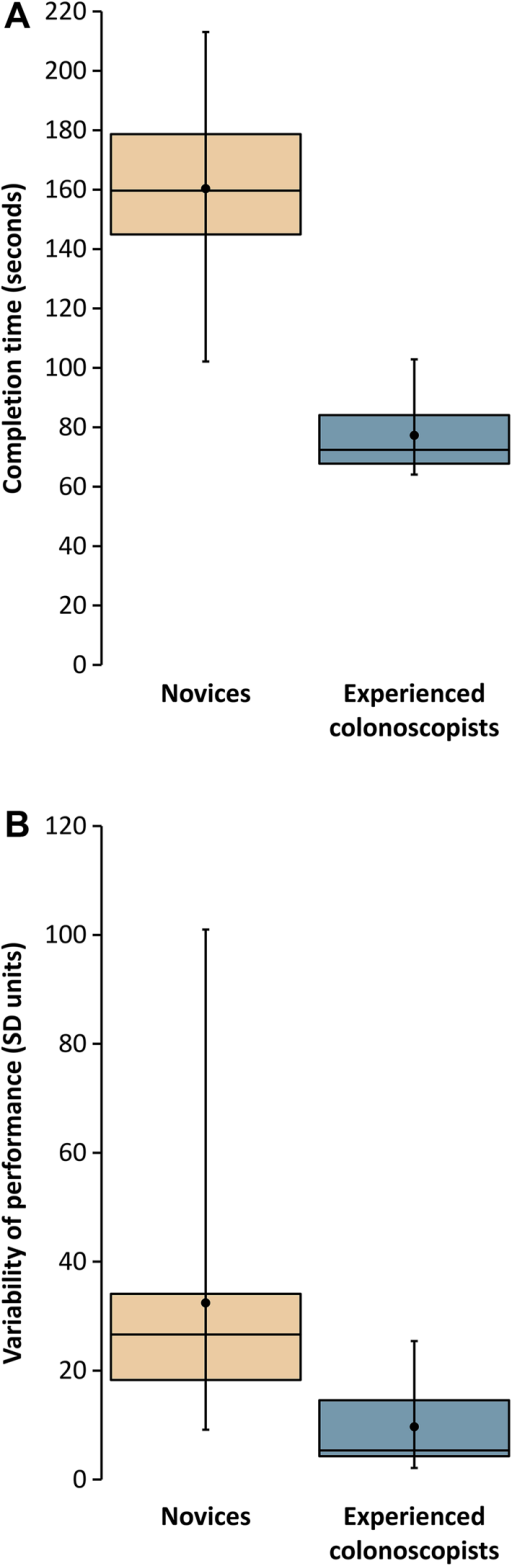
Validation study results (study 1). Box plots showing mean completion time (**A**) and variability of individual performance across trials (**B**) for each experience group. The *line inside each box* represents the sample median, and the *dot* represents the mean. The *boundaries of each box* represent the 25th and 75th percentiles, and the whiskers represent the minimum and maximum values

The *completion time* measure had excellent internal consistency reliability (α = 0.90). Figure 2 presents the *completion time* and *variability of individual performance* data for the two groups of participants. Compared with the novices, the experienced colonoscopists completed the training surfaces significantly faster, *U* = 1.00, *z* = −4.52, *P* < 0.0001, *r* = −0.84, and their performances were significantly less variable, *U* = 20.00, *z* = −3.69, *P* < 0.0001, *r* = −0.68.

### Training study (study 2)

**Fig. 3:**
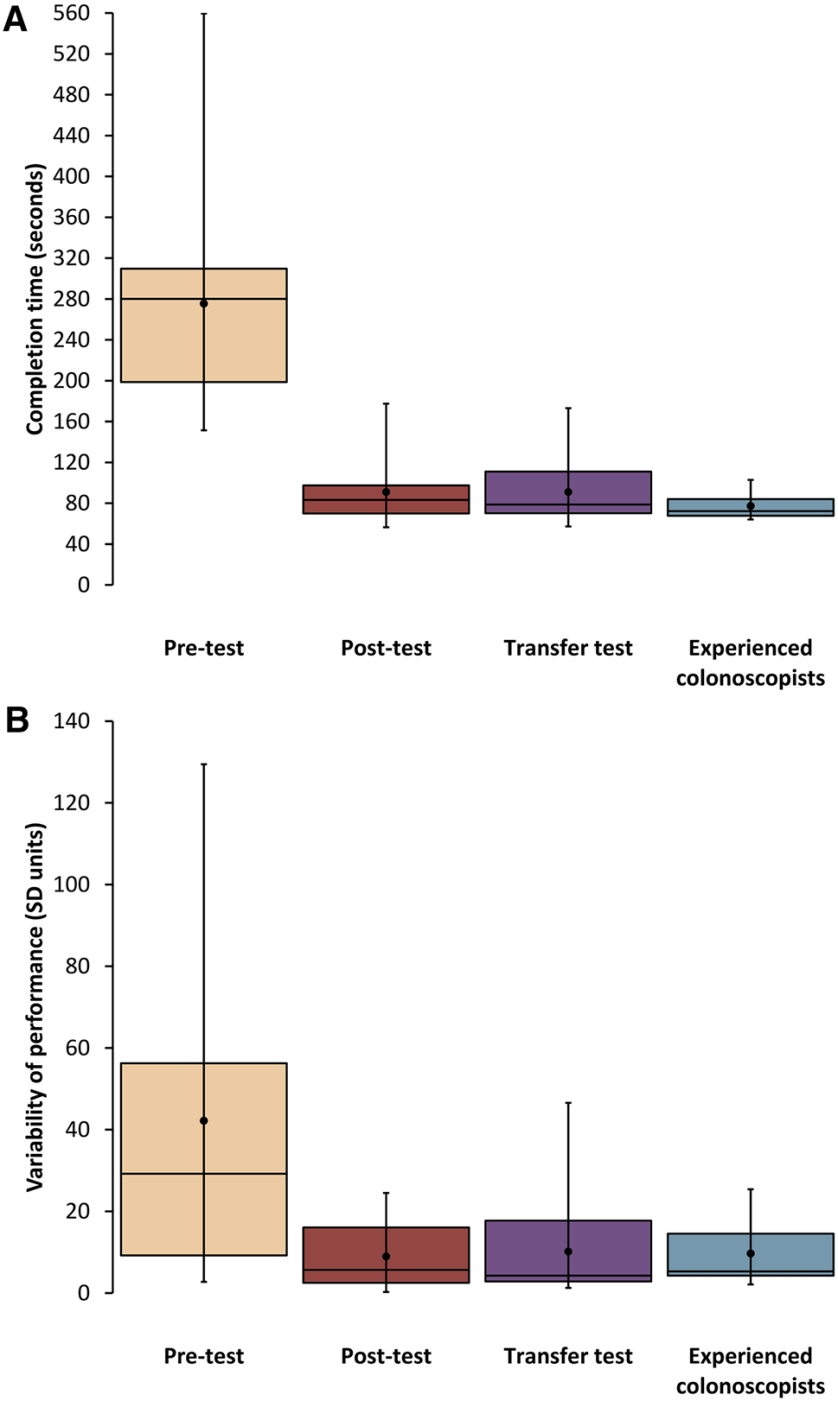
Training study results (study 2). Box plots showing mean completion time (**A**) and variability of individual performance across trials (**B**) for novices at pre-test, post-test, and transfer test. For comparison, equivalent data from the validation study experienced colonoscopist group are also included. The *line inside each box* represents the sample median, and the *dot* represents the mean. The *boundaries of each box* represent the 25th and 75th percentiles, and the *whiskers* represent the minimum and maximum values

The internal consistency reliability of the *completion time* measure was good-to-excellent for the pre-test (α = 0.88), post-test (α = 0.93), and transfer test (α = 0.88), and acceptable for the experienced colonoscopist data (α = 0.72). Figure 3 presents the *completion time* and *variability of individual performance* data for the three tests completed by the novice trainees: pre-test, post-test, and transfer test. It also compares these means with the equivalent experienced colonoscopist data from the preliminary validation study.

With training (pre-test vs. post-test), novice trainees became significantly faster, *T* = 0, *z* = −3.52, *P* < 0.0001, *r* = −0.62, and their performances significantly less variable, *T* = 14, *z* = −2.80, *P* = 0.003, *r* = −0.49. These improvements also generalized to novel training surfaces (pre-test vs. transfer test: completion time, *T* = 0, *z* = −3.52, *P* < 0.0001, *r* = −0.62; variability, *T* = 10, *z* = −3.00, *P* = 0.001 *r* = −0.53). In addition, the trainees’ performance on the transfer test was not significantly different from their post-test performance, either for completion time, *T* = 51, *z* = −0.88, *P* = 0.40, or variability, *T* = 67, *z* = −0.05, *P* = 0.98. Note that applying a “conservative” Bonferroni correction for multiple comparisons would not have affected the pattern of results reported here, and would arguably have been *less* conservative given that, for each outcome measure, we expected one of the three planned comparisons (i.e., post-test vs. transfer test) to be non-significant.

By the end of training, the performance of the novice group did not differ significantly from that of the experienced colonoscopists, either in terms of completion time, *U* = 73.00, *z* = −1.359, *P* = 0.19, or variability, *U* = 88.00, *z* = −0.70, *P* = 0.50.

## Discussion

This study provides preliminary evidence to support the validity of performance measures derived from using a novel tip control training device to assess skill, and the efficacy of the device as a training tool. Precise colonoscope tip control is essential for efficient intubation and mucosal inspection, as well as therapeutic procedures such as polypectomy [2, 3]. Despite the importance of this fundamental skill component, prior to the present study there was no reported validation evidence for a training regime specifically designed to aid its acquisition in isolation.

The data from the first of our two studies indicate that the performance measures derived from the device have sufficient measurement sensitivity to distinguish between experienced colonoscopists and novices in terms of their ability to control and guide the tip of the colonoscope quickly and accurately. Both of the performance measures (i.e., *completion time* and *variability of individual performance*) yielded preliminary evidence to support their construct validity: as we would expect, the experienced colonoscopists were significantly faster than the novices, and their performances were significantly less variable. These relationships between experience and performance are consistent with the task and metrics being a valid means of assessing colonoscopic tip control skill. It should also be noted that, although tip control is a psychomotor task, general psychomotor speed does not provide an alternative explanation for the results, given that the experienced colonoscopists were inevitably older than the novices and that psychomotor speed declines with age [23]. In addition, the composite measures of *completion time* used in both studies all had acceptable to excellent alpha reliability, indicating consistent internal structures and providing further validity evidence. These findings were the product of a response process deliberately designed to minimize the error associated with data collection, thus improving the quality and validity of the performance data. Specifically, the target acquisition software was engineered to require highly accurate tip maneuvers from all users, such that each target had to be precisely centered in the on-screen “target zone” for 25 ms before it was accepted as a valid acquisition and the user was allowed to continue on to the next target in the trail. In addition, the fact that the task involved using an actual colonoscope and endoscopy system, and required participants to precisely control and guide the colonoscope tip using the angulation controls and torque steering, suggests that its content is logically related to, and representative of, the task of tip control.

Furthermore, in the training study, novice participants significantly improved their performance on both outcome measures from pre- to post-test, having received six structured practice sessions in between. By the end of the training period, the novices’ performance on both metrics was similar to that of the experienced colonoscopists from the validation study. In fact, in our sample, there was no significant difference between the groups for either metric. This is arguably unsurprising given that colonoscopic tip control does not depend on esoteric clinical knowledge but relies primarily on fine motor skills, which can usually be acquired—and even mastered—relatively easily by most young adults, provided that they are motivated to learn [24]. However, a limitation of the study is that we cannot rule out the possibility that the small inter-group performance differences (which favored the experienced group) would have been statistically significant with a larger sample or a more sensitive test. Nevertheless, it not necessary for the novices’ post-test tip control performance to have reached a level entirely equivalent to that of the experienced colonoscopists in order for the training regime to be deemed successful and a potentially valuable precursor to training with live patients.

These results demonstrate the viability of using a part-task training device for novices to rapidly develop their tip control skill to a significantly improved level of proficiency. The only commercially available device that includes a task that can arguably be used to train tip control specifically is the GI Mentor II virtual reality colonoscopy simulator (Simbionix, Cleveland, USA). This device has a module requiring somewhat precise colonoscopic navigation in order to pop virtual bubbles arranged within a simulated colon (EndoBubble). Metrics derived from using EndoBubble have also been shown to distinguish between experienced and novice performance [25]. With practice, the performance of novices on this task improved significantly over the course of 4 sessions comprising 15 exercises in total, including virtual colonoscopy, but did not reach the level of experienced endoscopists [26].

Compared with the EndoBubble task, there are several advantages to using the novel device for initial tip control training. First, EndoBubble is not focused solely on tip control as it also requires the colonoscope to be inserted; hence, the task may not be as suitable for the complete beginner for whom simply using the angulation controls effectively, and integrating the use of torque, may be difficult enough. Second, compared with the targets in our novel task, the EndoBubble targets are relatively large and do not have to be targeted as precisely; hence, the same level of fine tip control is not required. Third, the cost of virtual reality simulators, such as the GI Mentor II, can be prohibitively expensive for wide distribution. Fourth, the modified colonoscope used in conjunction with the GI Mentor II has been rated by experienced colonoscopists as significantly less realistic than a genuine colonoscope [27].

In contrast, for a small fraction of the cost of a virtual reality simulator, the novel device presented here (or similar future devices, subject to validation) could be utilized with the existing equipment found in any endoscopy unit. Consequently, training programs such as ours could be made readily available for novice colonoscopists to acquire fine tip control skill (and perhaps also for clinicians who perform only occasional procedures, to maintain their proficiency). Indeed, our prototype device has already been used in Australian introductory endoscopy training workshops for surgical fellows, gastroenterology fellows, and nurse endoscopists. Training on the device has also been incorporated into a national curriculum for pre-clinical colonoscopy training, which has been successfully piloted for a nurse endoscopy program [28].

We have demonstrated that the tip control ability of colonoscopy novices can be improved dramatically in a short space of time using a low-cost targeted intervention. Given the fundamental importance of tip control, such interventions should form an integral part of any basic training program in colonoscopy. Trainees who achieve automaticity in tip control during the very earliest stages of their training should have more cognitive resources available to devote to other components of colonoscopy skill when additional complexities are introduced, either in higher fidelity simulations or in the procedure room. We predict that this is likely to lead to faster acquisition of more sophisticated skill components, and to improved patient safety during training lists. However, a substantial program of further research is required to demonstrate these flow-on effects. Another potentially fruitful avenue for future research might be to develop and validate modified devices and curricula that allow trainees to rehearse a broader range of tip control maneuvers, including those required for specific therapeutic procedures (e.g., biopsy, polypectomy, and endoscopic submucosal dissection). Validated tip control devices, such as the one described here, also allow for the tip control performance of any individual trainee to be quantified and compared against expected standards to evaluate competence and provide meaningful feedback.
